# Delayed Dorsalis Pedis Artery Pseudoaneurysm Following Open Reduction and Internal Fixation of a Lisfranc Injury: A Case Report

**DOI:** 10.7759/cureus.106848

**Published:** 2026-04-11

**Authors:** Yousif Jihad, Sedeek Mosaid, Ashok Marudanayagam

**Affiliations:** 1 Trauma and Orthopaedics, Lincoln County Hospital, United Lincolnshire Teaching Hospitals NHS Trust, Lincoln, GBR; 2 Orthopaedic Surgery, Lincoln County Hospital, United Lincolnshire Teaching Hospitals NHS Trust, Lincoln, GBR

**Keywords:** artery pseudoaneurysm, dorsalis pedis artery, lisfranc fracture, open reduction and internal fixation, reconstructive foot and ankle surgery

## Abstract

Vascular complications following operative fixation of Lisfranc injuries are rare and may present in a delayed manner. This report describes a case of a woman in her early 60s who sustained a Lisfranc fracture-dislocation following a fall from height and was treated with open reduction and internal fixation. Initial recovery was uncomplicated. The patient re-presented two months later with recurrent pulsatile bleeding from the dorsal midfoot wound and symptomatic anaemia. CT angiography revealed a 14 × 8 mm pseudoaneurysm arising from the dorsalis pedis artery adjacent to the fixation hardware. Diagnostic angiography confirmed thrombosis of the pseudoaneurysm with preserved distal arterial runoff. The patient was managed conservatively without surgical or endovascular intervention. This case highlights a clinically important but uncommon delayed vascular complication following Lisfranc fixation. It emphasises the role of early vascular imaging in patients presenting with delayed postoperative bleeding and demonstrates that conservative management may be appropriate in selected cases. Importantly, it underscores a decision-making paradigm in which intervention can be safely avoided when spontaneous thrombosis and adequate perfusion are present.

## Introduction

Lisfranc injuries are complex midfoot injuries that frequently require operative fixation to restore joint stability and alignment. While postoperative complications such as infection, hardware failure, and non-union are well recognised, vascular complications are rare and often underreported. In particular, pseudoaneurysm of the dorsalis pedis artery following Lisfranc fixation is an uncommon complication, with only a limited number of cases described in the literature [1,2].

The dorsalis pedis artery lies in close proximity to the tarsometatarsal joints and is therefore vulnerable during dorsal surgical approaches and hardware placement. Reported mechanisms include direct intraoperative injury, postoperative attritional damage from implants, and delayed vessel wall weakening. However, the true incidence of this complication remains unclear, and its delayed presentation may contribute to under-recognition.

Clinically, dorsalis pedis artery pseudoaneurysms may present with non-specific or delayed symptoms, including swelling, pain, pulsatile bleeding, or anaemia, which can mimic more common postoperative complications such as infection or haematoma. This creates a diagnostic challenge, particularly in the absence of early vascular imaging.

Various management strategies have been discussed, including surgical ligation, endovascular embolisation, and ultrasound-guided thrombin injection, but there is no clear gold standard for optimal management, particularly in cases where spontaneous thrombosis occurs and distal perfusion is preserved. Decisions are often individualised, with limited guidance from existing literature.

Delayed pseudoaneurysm formation following internal fixation can present insidiously and may be overlooked [3,4]. This case illustrates a rare late complication after Lisfranc surgery and underscores the importance of vigilance where patients present with delayed bleeding or wound complications.

## Case presentation

A woman in her early 60s presented following a mechanical fall from a ladder, landing on her left foot. She reported immediate pain, swelling, and inability to bear weight. Clinical examination revealed swelling and tenderness over the dorsal midfoot with intact neurovascular status.

**Figure 1:**
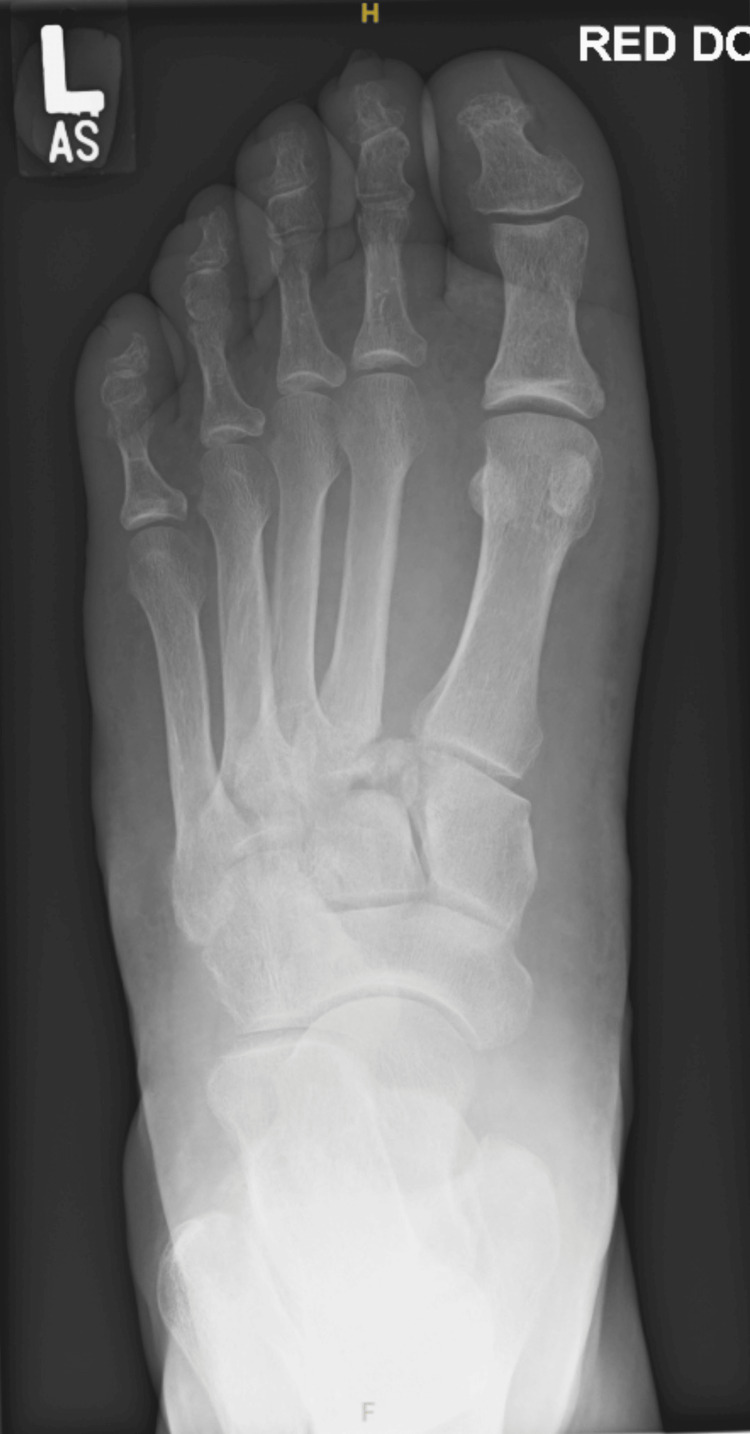
Plain radiograph of the left foot demonstrating widening of the first-second intermetatarsal space with a displaced fracture at the base of the second metatarsal, consistent with a Lisfranc injury.

**Figure 2:**
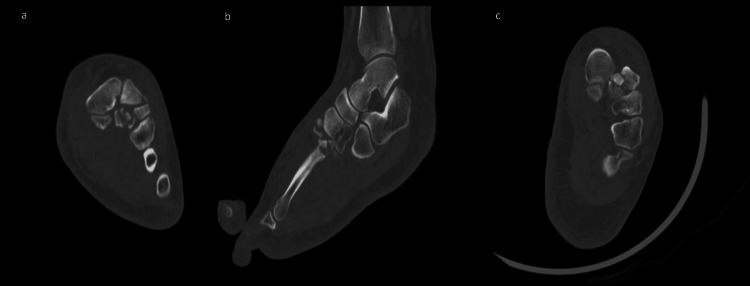
CT images of the left foot (a: axial, b: sagittal, c: coronal) demonstrating a comminuted intra-articular fracture at the base of the second metatarsal with widening of the first-second intermetatarsal space, confirming an unstable Lisfranc fracture-dislocation.

Initial plain radiographs demonstrated a displaced intra-articular fracture at the base of the second metatarsal with widening of the first-second intermetatarsal space, suspicious for Lisfranc injury (Figure 1). Subsequent CT of the foot confirmed a laterally displaced comminuted intra-articular fracture of the second metatarsal base, associated avulsion fracture of the medial cuneiform, and widening of the proximal interosseous space, consistent with an unstable Lisfranc fracture-dislocation (Figure 2).

**Figure 3:**
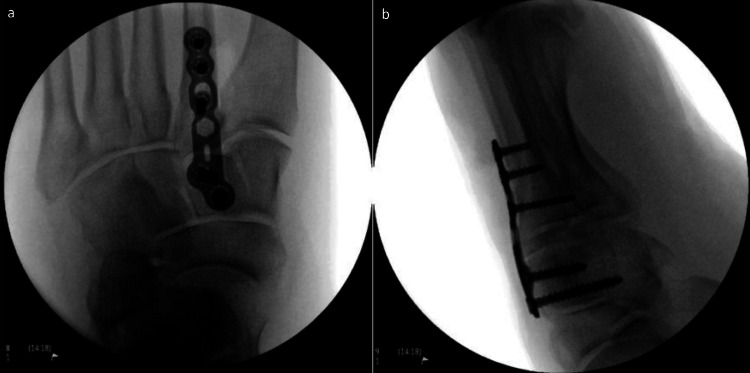
Intraoperative fluoroscopic images demonstrating dorsal plate and screw fixation across the tarsometatarsal joints following open reduction and internal fixation of the Lisfranc injury. a: Anteroposterior intraoperative fluoroscopic view demonstrating dorsal plate fixation across the tarsometatarsal joints. b: Lateral intraoperative fluoroscopic view demonstrating screw positioning and alignment following fixation.

The patient underwent open reduction and internal fixation using dorsal plate and screw fixation (Figure 3). Postoperative recovery was initially uneventful; the patient was discharged non-weight bearing with routine follow-up.

The patient re-presented approximately two months later with intermittent pulsatile bleeding from the dorsal surgical wound, associated with increasing pain and swelling. Examination demonstrated active bleeding episodes with preserved distal perfusion and intact sensation. Laboratory investigations revealed a significant reduction in haemoglobin, requiring red blood cell transfusion.

**Figure 4:**
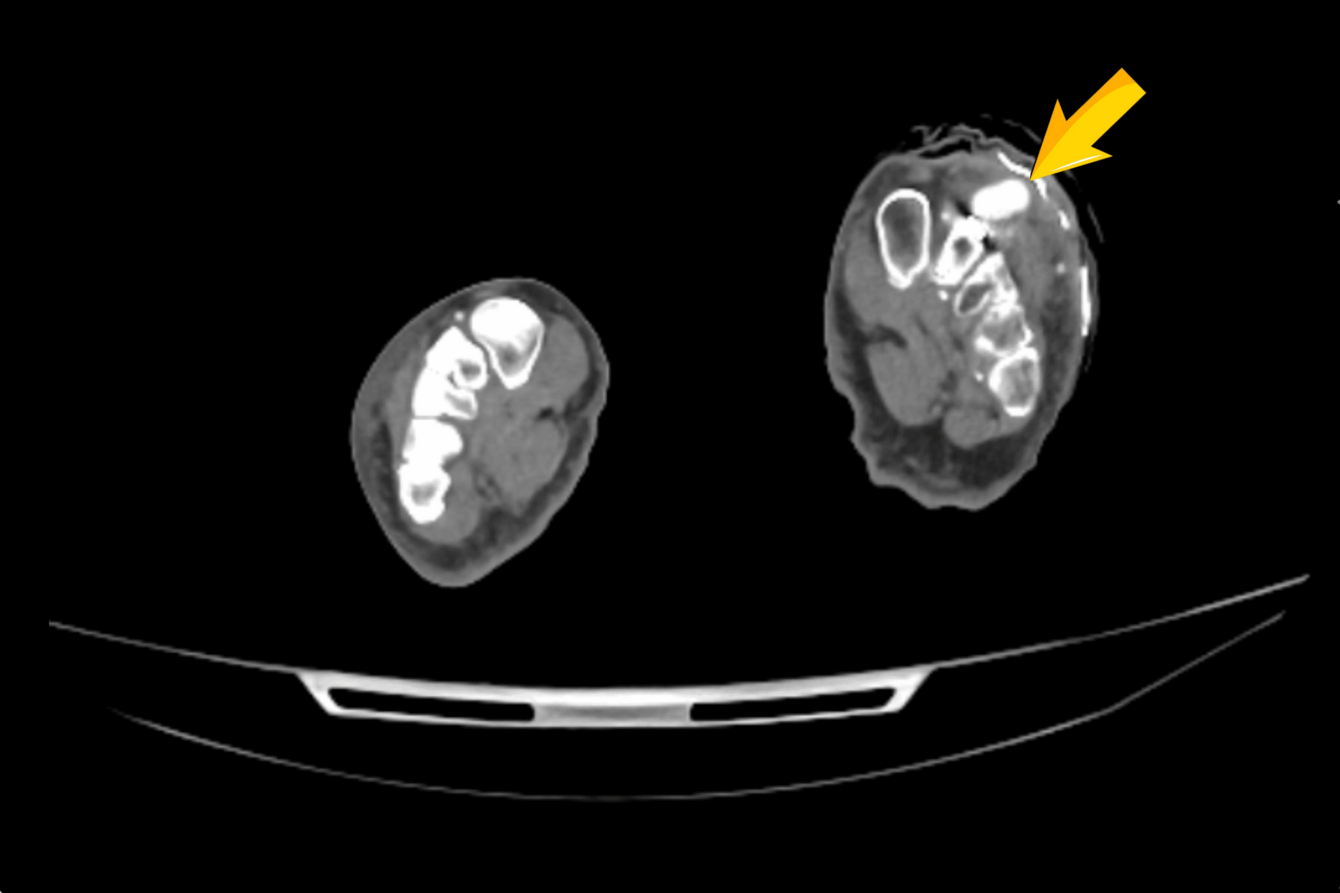
Axial CT angiography of the left foot demonstrating a well-defined contrast-filled outpouching (yellow arrow) arising from the dorsalis pedis artery at the level of the tarsometatarsal joints, consistent with a pseudoaneurysm.

CT angiography of the lower limbs identified a well-defined contrast-filled outpouching (measuring approximately 14 × 8 mm) arising from the dorsalis pedis artery at the level of the tarsometatarsal joints adjacent to the fixation hardware, consistent with a pseudoaneurysm and surrounding soft tissue oedema (Figure 4).

**Figure 5:**
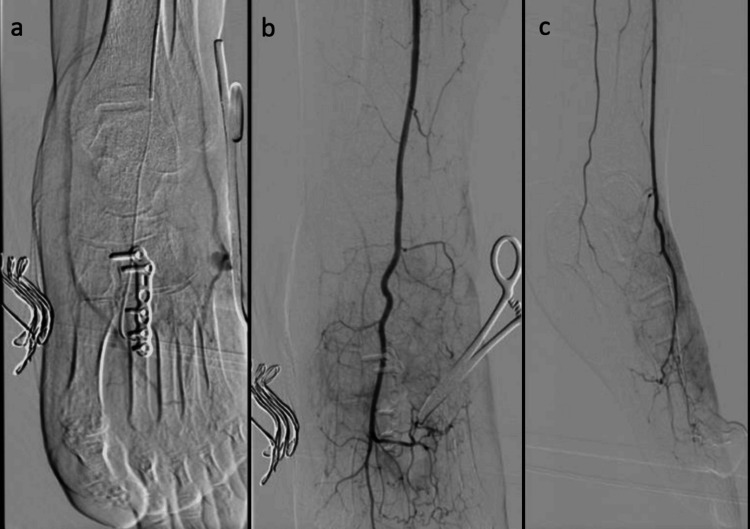
Selective angiography of the left dorsalis pedis artery. a: Anteroposterior projection demonstrating fixation hardware and regional vascular anatomy. b: Magnified anteroposterior projection highlighting the dorsalis pedis artery at the level of the pseudoaneurysm. c: Oblique projection demonstrating thrombosis of the pseudoaneurysm with preserved distal arterial runoff and no active contrast extravasation.

Diagnostic angiography showed patent femoral, popliteal, and infrapopliteal vessels with preserved triphasic runoff. Selective imaging of the dorsalis pedis artery confirmed thrombosis of the pseudoaneurysm without active contrast extravasation and no internal flow (Figure 5).

No endovascular or surgical intervention was performed as haemorrhage had ceased and distal perfusion remained adequate. The patient was managed conservatively with wound care, haemoglobin optimisation, and close multidisciplinary follow-up. No further bleeding episodes occurred, and recovery progressed without additional complications.

## Discussion

Pseudoaneurysm of the dorsalis pedis artery is an uncommon complication [1-3] most frequently associated with penetrating trauma or iatrogenic injury [3,4]. Its superficial course over the tarsometatarsal joints places it at risk during surgical approaches for Lisfranc fixation, particularly where dorsal plating is employed [1-3].

From a surgical perspective, this case also highlights the anatomical vulnerability of the dorsalis pedis artery during dorsal approaches to the tarsometatarsal joints [1]. The artery typically courses superficial to the joint capsule and hardware trajectory, rendering it susceptible to both direct intraoperative injury and delayed attritional damage from prominent implants or postoperative micromotion [2,3]. While meticulous soft tissue handling and awareness of vascular anatomy are standard practice, even technically uncomplicated procedures may result in occult arterial injury manifesting only after wound healing and resumption of activity. This underscores the importance of maintaining a high index of suspicion where patients present with unexplained postoperative bleeding, swelling, or anaemia following midfoot surgery. In such scenarios, early cross-sectional vascular imaging may prevent repeated haemorrhage and avoid potentially catastrophic outcomes.

Further, this case supports the role of multidisciplinary collaboration between orthopaedic surgeons, vascular surgeons, and interventional radiologists in determining optimal management. Individualised decision-making is essential, particularly when spontaneous thrombosis has occurred and distal perfusion is preserved, as unnecessary intervention carries its own risk. Incorporating awareness of delayed vascular complications into postoperative follow-up and patient education may facilitate earlier recognition and prompt investigation in future cases.

Delayed presentation, as observed in this case, may result from gradual arterial wall injury, hardware irritation, or postoperative wound breakdown [4]. Clinical features include pulsatile bleeding, expanding mass, pain, or anaemia and may manifest weeks to months following surgery [3,4]. Awareness of this complication is essential, as delayed diagnosis can result in significant haemorrhage.

CT angiography facilitates accurate localisation and characterisation of vascular injury and should be performed promptly where vascular complications are suspected [5]. Management strategies include surgical ligation, endovascular embolisation, ultrasound-guided thrombin injection, or conservative management in cases of spontaneous thrombosis with preserved collateral circulation [6,7]. In this case, conservative management was appropriate due to spontaneous thrombosis and preserved distal perfusion.

## Conclusions

Delayed dorsalis pedis artery pseudoaneurysm is a rare but potentially serious complication following open reduction and internal fixation of Lisfranc injuries that may present weeks after an initially uncomplicated postoperative course. This case highlights the importance of maintaining a high index of suspicion in patients presenting with delayed postoperative bleeding, swelling, or unexplained anaemia. Early use of vascular imaging, particularly CT angiography, is essential for accurate diagnosis and appropriate management.

While CT angiography plays a central role in diagnosis, management should be guided by haemodynamic status, pseudoaneurysm characteristics, and distal perfusion. Conservative management may be a safe and effective option in selected cases where spontaneous thrombosis has occurred and distal perfusion is preserved. Ultimately, this case emphasises the need for careful surgical technique, awareness of regional vascular anatomy, and multidisciplinary collaboration to optimise patient outcomes and minimise complications.
